# Mothers of infants and toddlers: basic markers of psychological well-being and their correlation with optimism

**DOI:** 10.1192/j.eurpsy.2025.2403

**Published:** 2025-08-26

**Authors:** E. V. Deshchenko, E. V. Polonetskaya, E. I. Pervichko

**Affiliations:** 1 Lomonosov Moscow State University, Moscow, Russian Federation

## Abstract

**Introduction:**

Psychological well-being of women during the first years of their maternity is essential for both them and their families. However in the same time mothers of infants and toddlers face a lot of challenges in fulfilling their role so their well-being should be closely monitored.

**Objectives:**

The research aimed to study the basic markers of the psychological well-being of mothers with little children, such as depressive tendencies, anxiety, optimism and aggression.

**Methods:**

To measure depressive tendencies the Beck Depression Inventory (as adapted by N.V. Tarabrina) was used. Anxiety levels were assessed using the State-Trait Anxiety Inventory by C. Spielberger (as adapted by Yu.L. Khanin). Life Orientation Test (as adapted by T.O. Gordeeva *et al* ) was used to assess respondents’ optimism levels and Bass-Perry Aggression Questionnaire was used to assess the aggression factors (as adapted by S.N. Enikolopov and Tsybul’skii N.P.). Spearman’s rank correlation coefficient was used for data analysis. The research was conducted from September 2023 to August 2024. The sample consisted of 253 mothers and 31 fathers (as the control group) with children of three years old and less.

**Results:**

Our sample demonstrated comparably high levels of anxiety and average levels the aggression factors and optimism for women as set out in the table below (mean scores shown) (table 1).

Table 1. Comparative analysis of psychological well-being in women and men.

**Table:** 

	*State anxiety*	*Trait anxiety*	*Anger*	*Hostility*	*Physical aggression*	*Optimism*
***Women***	48±24.53%	49±23.48%	20±26.97%	21±31.53%	20±32.22%	17±32.70%
***Men***	41±30.58%	42±27.12%	17±26.97%	19±31.53%	22±32.22%	17±32.70%

Results of 73 women (29%) show expressive depressive tendencies and results of 67 women (27%) show severe depression tendencies (using the cut-off points proposed by Andriushchenko A.V. and his colleagues (Andriushchenko *et al*. Zh Nev rol Psikhiatr Im S S Korsakova 2003; 103(5) 11–18 ), being 12 points for the expressed depressive tendencies and 20 points for the severe depressive tendencies). Only results of 2 men from the control group (7%) show expressive depressive tendencies and results of 3 men (10%) show severe depression tendencies.

Negative correlations of the optimism levels of mothers to the depressive tendencies (r=-0.342, p=0.003) and hostility (r=-0.259, p=0.027) levels were discovered.

**Conclusions:**

Thus, the results of our study indicate that the depression and anxiety levels of mothers of infants and toddlers are comparably high, while the aggression and optimism indicators stay on average levels. The study also shows negative correlations of the optimism level of mothers with the depression tendencies and the hostility level.

**Disclosure of Interest:**

None Declared

